# Alcohol binge drinking in adolescence and psychological profile: Can the preclinical model crack the chicken-or-egg question?

**DOI:** 10.3389/fpsyt.2022.996965

**Published:** 2022-09-09

**Authors:** Valentina Castelli, Fulvio Plescia, Giuseppe Maniaci, Gianluca Lavanco, Giuseppe Pizzolanti, Anna Brancato, Carla Cannizzaro

**Affiliations:** ^1^Department of Biomedicine, Neurosciences and Advanced Diagnostics, University of Palermo, Palermo, Italy; ^2^Department of Health Promotion, Mother and Child Care, Internal Medicine and Medical Specialties, University of Palermo, Palermo, Italy

**Keywords:** alcohol binge drinking, late adolescence, drinking motives, psychopathology, resilience, self-esteem

## Abstract

During adolescence, internal and external factors contribute to engaging with alcohol binge drinking (ABD), putting at risk the neurodevelopment of brain regions crucial for emotional control and stress coping. This research assessed the prevalence of ABD in late adolescent students of Southern Italy and characterized their psychological profile and drinking motives. Translational effects of alcohol binge drinking in the animal model were also studied. Seven hundred and fifty-nine high school students of both sexes (aged 18–20) were recruited. Alcohol Use Disorder Identification Test-Consumption (AUDIT-C), Drinking Motives Questionnaire-Revised Short Form, Millon Clinical Multiaxial Inventory-Third Ed., State-Trait Anxiety Inventory, Connor-Davidson Resilience Scale, and Basic Self-Esteem Scale identified alcohol habits, drinking motives, and psychopathological profile. Eighty-five percentage of the students drank alcohol and 28% of them engaged in ABD; AUDIT-C correlated with enhancement, coping, and conformity motives. ABD was related to a greater likelihood of presenting clinical syndromes and personality disorders, as well as low resilience and self-esteem. Thereafter, in the pre-clinical model, adolescent male rats were exposed to alcohol (3.5 g/kg) in an intermittent binge-like paradigm and tested during prolonged abstinence. Rats were evaluated for anxiety-like behavior, motivated behaviors, resilience, and stress response following a psychosocial challenge. Binge-like alcohol-exposed adolescent rats displayed high integrated z-score for social- and novelty-induced anxiety, altered motivation-driven output, decreased resilience, and a blunted HPA axis response to psychosocial stress, with respect to respective controls. Our data confirm that ABD is the chosen pattern of drinking in a significant percentage of high school students in Southern Italy, and highlights AUDIT-C score as a relevant parameter able to predict the occurrence of affective disturbances. The evidence from the preclinical model shows that ABD produces detrimental consequences in the adolescent rat brain, resulting in negative affect, emotional dysregulation, and aberrant stress response, pointing to decreasing excessive alcohol drinking as a primary goal for the global act for brain health.

## Introduction

Drinking habits in European countries are shaped by culture, tradition and social-environmental influences. The “Nordic-pattern”, common in Scandinavia, United Kingdom, and continental Europe, relies on episodic heavy drinking of alcoholic beverages such as beer and spirits, while alcohol drinking habits in the Mediterranean countries have been traditionally characterized by drinking wine during meals (1). However, the “Nordic” drinking style is spreading among the Mediterranean “Millennials”, who increasingly indulge in drinking alcohol out-of-meal at intoxicating amounts (2). This pattern of consumption, namely alcohol binge drinking (ABD) occurs when females and males consume, respectively, 4+ and 5+ drinks in about 2 h and blood alcohol concentration reaches 0.08 g/dl (3).

One of the reasons for this conforming trend throughout Europe, besides changes in social and cultural factors that are not currently addressed, may lie in drinking motives, that have emerged as strong predictors of adolescent alcohol use (4). They are assumed to mediate the subjectively-derived decisional framework for alcohol (mis)use, that includes personality features, culture-specific drinking styles, and situational factors (5, 6). The Motivational Model by Cox and Klinger (7) is one of the main theoretical frameworks for the investigation of alcohol consumption, which postulates that motivations for drinking alcohol are based on the valence (positive or negative) and the source (internal or external) of the desired outcomes. Hence, individuals drink to obtain positive outcomes or to avoid negative consequences, and they are motivated by internal or external rewards. Accordingly, four categories of drinking motives emerge as final antecedents of drinking behavior: 1. enhancement: to heighten mood or wellbeing (internally generated with positive valence); 2. social: to obtain social rewards (externally generated with positive valence); 3. coping: to attenuate negative emotions (internally generated with negative valence); 4. conformity: to avoid social rejection (externally generated with negative valence) (8).

Besides, drinking motives can shape drinking patterns, with enhancement and coping motives being the most correlated with ABD (4, 8–10). The re-enactment of the determinant framework appears to be crucial to the evaluation of the concerning consequences in adolescence, given the “still-going-on” maturation of the brain and the major vulnerability to alcohol during neurodevelopment (11, 12). Indeed, functions highly relevant for successful adaptation to peculiar social demanding contexts—cognitive functioning, affective processes, and emotional control—which require unique biological maturation and social development, may be jeopardized by ABD (13–15). In turn, maladaptive coping to social challenges produces an aversive emotional state which may promote alcohol misuse, thus triggering a fly-wheel for the development of neuropsychiatric disorders (16, 17).

Several studies on the general and clinical populations have shown how alcohol abuse can precede the onset of clinical syndromes, such as depression, anxiety, and personality disorders (18–21). On the other hand, the existence of certain psychopathological profiles characterized by anxiety, depression and low self-esteem may, in turn, influence the trajectory and pattern of alcohol consumption thus facilitating alcohol abuse (22–24). When alcohol misuse and psychiatric pathologies or personality disorders come together is what is usually called dual pathology; this represents an unanswered challenge in terms of the aetiological hypothesis (25, 26).

Notably, resilience is related to the mitigation of experienced aversive events, by providing the individual with the ability to adapt to stressful circumstances while maintaining mental wellbeing (27). In this scenario, resilience appears as a protective factor associated with a reduced risk of consuming alcoholic beverages (28, 29).

On this basis, our research aimed at taking a picture of alcohol drinking habits and related motives in high school students in Southern Italy, a region known for its strong familiar, and traditional hard-core. The students were also assessed for personality disorders and clinical syndromes, as well as for levels of resilience and self-esteem, in order to explore the correlation between ABD and specific psychopathological profiles and estimate the probability of their occurrence according to the drinking pattern.

In clinical studies, the causal relationship between psychopathological profile and ABD is inconsistently proved and not totally supported by the temporal relationship between the onset of one and the occurrence of the other, thus hindering the address of specific intervention. Therefore, in an attempt to crack the chicken-or-egg question, we exploited the animal model, which allows analyzing complex pathological constructs by isolating their single behavioral features and studying their determinants. We explored the relationship between the exposure to a binge-like alcohol paradigm in adolescence, known to induce multilevel maladaptive plasticity (30) and the development of a vulnerable endophenotype for emotional dysregulation and negative affective state in the rat. In detail, we investigated ethologically-relevant behavioral and endocrine responses to emotionally-salient environmental stimuli, such as novelty and social stress as they represent common challenges adolescents face. These results will help to highlight ABD as a significant hindrance to the correct development of the neuropsychological capabilities of adolescent drinkers and as a major target for prevention.

## Materials and methods

### Drinking patterns, psychopathological profiles, motives in high school students

#### Participants and study design

A total of 759 of both sexes students—18–20 years—were enrolled from eleven high schools in Palermo and participated in the questionnaire-based study administered in school classrooms. Students with severe medical illnesses and use of illicit drugs were excluded. Signed informed consent was obtained from all the participants. The present work has been carried out in accordance with The Code of Ethics of the World Medical Association Declaration of Helsinki: ethical principles for medical research involving human subjects. The procedures were further approved by Ethics Committee Palermo 1.

#### Measures

##### Evaluation of alcohol drinking patterns and motives, and psychopathological profiles

###### Alcohol use disorder identification test-consumption

The Italian version of AUDIT-C was employed to identify alcohol drinking patterns – moderate-, binge-drinking, and abstention. AUDIT-C is based on the first three items of AUDIT and refers to the frequency of alcohol consumption, the number of standard drinks consumed on a typical day when drinking, and the frequency of consuming six or more standard drinks on one occasion. AUDIT-C uses a 5-point Likert scale, resulting in an overall score ranging from 0 to 12: scores of 0 were defined as current abstainers; scores <3 are consistent with non-at-risk alcohol consumption, thus moderate-drinkers scored 1–2 (females) and 1–3 (males); binge-drinkers were defined as AUDIT-C scores of ≥3 (females) and ≥4 (males). Previous studies have confirmed internal consistency α = 0.75 (31).

###### Drinking motives questionnaire-revised short form

Students' motives to engage in alcohol drinking were explored by the 12-item Italian version of the DMQ-R-SF. Each item is a statement concerning the frequency of drinking for four distinct dimensions (enhancement, social, conformity, and coping). Participants were asked to consider, whether they have drunk alcohol in the last 12 months, to indicate on how many occasions they have drunk for each given motive. Each dimension consists of three items and is rated on a frequency scale ranging from “Never” (coded as 1) to “Almost always” (coded as 3). The Italian version of the instrument was previously validated (α = 0.64–0.79) (32).

###### Millon clinical multiaxial inventory-third ed

To assess personality disorders and clinical syndromes, we employed the Italian third version of MCMI. MCMI-III is a 175-item true/false self-report instrument that identifies 14 personality disorder- and 10 clinical syndrome scales. The MCMI-III raw scores are reported as weighted base rate scores. Validity scales were used to provide information about the students' response styles, in order to detect and invalidate random responding. The scales were previously validated (α = 0.66–0.90; test-retest reliability *r* = 0.84–0.96) (33).

###### State-trait anxiety inventory (STAI-S/T)

The 40-item Italian version of STAI form Y consists of two subscales—State (STAI-S) and Trait (STAI-T), each one composed of 20 items—which were employed to, respectively, measure both transitory/present- and stable anxiety. Responses are given on a 4-point Likert scale (from 1 “Not at all” to 4 “Very much so”). Total scores range from 20 to 80 for each subscale. The Italian version of the instrument was previously validated (α = 0.91–0.95) (34).

###### Connor-Davidson resilience scale (CD-RISC-25)

The Italian validated version of the scale was employed as a measure of resilience which includes 25 items, scored on a 5-point Likert scale −0 = Totally disagree, 4 = Totally agree. The total score ranges from 0 to 100, with higher scores meaning higher resilience. The internal consistency of the scale was previously confirmed by α = 0.93 (35).

###### Basic self-esteem scale

The scale assesses basic self-esteem and includes 22 items, scored on a 5-point Likert scale – 0 = Totally disagree; 4 = Totally agree. The Italian version of the instrument was previously validated (α = 0.85; test-retest reliability = 0.81–0.83) (36).

###### Substance co-use

Tobacco use and cannabis consumption were categorical variables with two response options with a value of 1 for “smoker” and a value of 0 for “non-smoker” (37).

### Preclinical model of binge-like alcohol exposure

#### Animals

Male Wistar rats (Envigo, Italy) arrived on postnatal day (PND) 21 and were housed in pairs in standard polycarbonate cages with standard bedding. Male retired breeder Wistar rats from Envigo (Italy), employed as aggressors in the social stress procedure, were single-housed in standard polycarbonate cages filled with standard bedding. All rats were maintained at the temperature of 22 ± 2°C, with 55 ± 5% humidity, on a 12 h light/dark cycle (lights on at 08:00 a.m.). Laboratory rodent chow (Mucedola, Italy) and tap water were available *ad libitum*. Procedures were approved by the Italian Ministry of Health (1119/2016-PR), in adherence with the current Italian regulation (D.L. 26/2014) on laboratory animals care and use and EU Directive 2010/63/EU for animal experiments. Every effort was made to minimize the number of animals used and their suffering.

#### Anxiety-like phenotype and stress reactivity evaluation in a rat model of binge-like alcohol exposure

##### Preclinical model of binge-like alcohol exposure

Rats were randomly assigned to two experimental groups (*n* = 7 per group): alcohol naïve (CTRL) rats and binge-like alcohol-exposed (BAW) rats. BAW rats were exposed to alcohol in an intermittent binge-like alcohol paradigm during adolescence (PND 35–54). The 25% alcohol solution was daily prepared and administered per os (38), at the dosage of 3.5 g/kg (39), 3 days a week, every other day, for a total of 9 exposure, as previously described (30). CTRL rats were given an isovolumetric amount of tap water on the same exposure days. All PND 21 rats were gently handled and habituated to the oral administration procedure from PND 28 onwards. Rats were gently administered with alcohol (or water) by introducing the calculated amount of solution in the rat's mouth through a laboratory pipette. This procedure was aimed at decreasing the distress of gavage in adolescent rats, employing the common administration route of alcohol consumption in humans, and ensuring accurate dosing. In the same experimental conditions, binge-like alcohol-exposed rats displayed a blood alcohol concentration of 193 ± 19 mg/dl when sampled 1 h after the administration on the last binge-like alcohol exposure, indicating intoxicating binge-like blood alcohol levels (>80 mg/dl) (30). After 10 days from the last administration, CTRL and BAW rats were tested for anxiety-like phenotype and stress response as described below.

##### Social interaction test

The social interaction test (SI) has been used extensively for the assessment of anxiety-like behavior in laboratory rodents (39, 40). Here we employed a 2-compartment testing apparatus, in order to assess anxiety-like behavior under social circumstances, in terms of social avoidance, and differentiate from confounding behavioral categories (e.g., social investigation and play fighting) (30). On the test day, rats were individually placed in the plexiglass testing apparatus (45 × 45 × 30 cm) divided into two equally sized compartments by a partition, which allowed free movements between the two halves, for 2 min. A social “stimulus” male rat, of the same age and similar weight as the experimental rat, was then introduced for the 10-min test period. Stimulus rats were always unfamiliar with the experimental rat and experimentally naive. The test sessions were recorded by a video camera and the time spent in the compartments, either occupied or not by the stimulus rat, was measured by a trained experimenter, blind to the treatment, using Boris v. 7.9.4. Exploratory behavior was controlled in terms of the number of crossovers between the two compartments during the 2-min habituation. To recapitulate the anxiety-like phenotype, leveraging potential biases induced by a single test, an integrated *z*-score was calculated as follows *z* = (X–μ)/σ, indicating how many standard deviations (σ) the observation (X) is above or below the mean of the control group (μ) (41) based on the SI, using normalization of social avoidance values, i.e., the percentage of time the rat spent in the compartment unoccupied by the social stimulus rat. Individual anxiety-like z-scores were then calculated by averaging *z*-score values.

##### Novelty suppressed feeding test

Rats were food restricted overnight prior to the novelty suppressed feeding test (NSFT) and habituated to the testing room for 1 h on the test day. Under dim light conditions, rats were then placed into a plastic box 50 × 50 × 20 cm with bedding, where a single pellet of food was placed in the center. Rats were placed in the corner of the box, and the latency to eat was scored up to 10 min during testing by a trained observer, blind to the treatment. Immediately afterwards, the rat was transferred to the home cage, where the latency to eat was timed, serving as a control for the change in appetite as a possible confounding factor. The integrated *z*-score was calculated based on the NSFT, using normalization of the latency time to eat the pellet. Individual anxiety-like *z*-scores were then calculated by averaging *z*-score values.

##### Forced swimming test

The modified forced swimming test (FST) here employed was previously described by Cryan et al. (42). In this single-session test, rats were placed individually in clear cylinders (40 cm high, 18 cm inside diameter) filled with 5–6 l of clean water at 22–23°C, for 15 min. The sessions were videotaped for subsequent analysis, performed by a trained experimenter blind to the treatment, using Boris v. 7.9.4. The duration of swimming, defined as active movements of the rat's four paws during the first 5 min, was recorded as a measure of proactive coping with the swim stress, and control-mean-normalized values were calculated as a swim stress resilience z-score.

##### Neuroendocrine response to social stress exposure

Separate cohorts of CTRL and BAW rats (*n* = 7 per group) were exposed to the social stress paradigm to assess their Hypothalamic-Pituitary-Adrenal (HPA) axis reactivity compared with no-stress exposed counterparts (*n* = 7 per group), who remained undisturbed in their home cage during the same days. Rats underwent the social stress procedure on three separate days, as previously published (43). Briefly, we used a clear Plexiglas box (45 × 45 × 30 cm) divided into two equal chambers with a plastic wall. Here, stress-exposed rats were confined within a metal cage (10 × 10 × 10 cm) in the middle of one chamber, while a retired breeder male Wistar, previously identified as aggressive in the confrontation with nonexperimental adolescent rats, was placed in the empty chamber, for 30 min per session. The aggressor rat was able to move freely around the testing apparatus, including up to the metal cage confining the social-stress exposed rat, but physical contact between the rats could not occur. The chambers and metal cages were thoroughly cleaned between each stress procedure. After the social stress paradigm, rats were sacrificed, and trunk blood samples were collected at early afternoon (1:00–3:00 p.m.). Serum was prepared according to standard protocols and kept at −20°C until the time of assay, when corticosterone levels (CORT, ng/ml) were measured using a commercially available ELISA kit (Demeditec), according to the manufacturer's instructions. Normalized values to no stress-exposed controls' mean level were used to calculate the neuroendocrine stress response *z*-score.

### Statistical analysis

The difference between groups' scores was determined by employing non-parametric Mann-Whitney test and Kruskal-Wallis one-way analysis of variance (ANOVA) when appropriate. Descriptive Statistics and Chi-Squared tests were performed to assess and compare prevalence. The STAI-S/T-, CD-RISC-25- and BASIC-SE scores were dichotomized by median split into high and low categories and Simple Logistic Regression was employed to predict the binary variables using AUDIT-C score as the independent variable, with the factor β1 referring to the change in binary variables when the variable AUDIT-C change one unit. All analyses were controlled for sex, age, and self-reported health status. Principal component analysis (PCA) was used to examine patterns of intercorrelations between the variables studied, as previously described (30). Here, the original datasets of each individual student containing 9 variables, including four parameters related to the psychopathological profile (STAI-S, STAI-T, CD-RISC-25, BASIC-SE) and five variables related to alcohol drinking pattern (AUDIT-C, DMQ-R-SF-Enhancement, DMQ-R-SF-Social, DMQ-R-SF-Conformity, DMQ-R-SF-Coping), were analyzed to obtain their correlation matrix and PCAs. The number of animals employed in preclinical experiments was calculated using an a priori power analysis based on effect sizes observed in our previous published work (30). *Z*-scores were analyzed using the Mann-Whitney test and two-way ANOVA (2-way ANOVA). Statistical analysis was performed using Prism v.9 (Graphpad). Data are reported as mean ± SEM and statistical significance was set at α = 0.05.

## Results

### Evaluation of alcohol drinking patterns and motives, and psychopathological profiles

Among total students, 84.59% of students, drink alcohol and 27.88% engage in ABD (Table 1). No significant differences in alcohol pattern consumption were found among sexes (χ^2^ = 5.127, df = 2, *p* = 0.0770). Besides, Chi-Squared test did not detect significant differences in the prevalence of tobacco use (abstainers vs. binge drinkers: χ^2^ = 0.9026, *p* = 0.3421; abstainers vs moderate drinkers: χ^2^ = 0.09860, *p* = 0.7535) and cannabis consumption (abstainers vs. binge drinkers: χ^2^ = 2.121, *p* = 0.1453; abstainers vs moderate drinkers: χ^2^ = 0.2086, *p* = 0.6479).

**Table 1:** Descriptive statistics of alcohol consumption patterns among high school students according to the AUDIT-C score.

**Alcohol consumption patterns**				
	***n***	**%**	**Audit-C score**	**95% CI**
			**average index**	
**Total students**	759	100		
Abstainers	117	15.41	–	–
Moderate-drinkers	463	61.01	1.754	1.691–1.816
Binge-drinkers	179	23.58	4.564	4.348–4.789
**% drinking pattern**				
Drinkers	642	84.59	2.537	2.414–2.661
Moderate	463	72.19	1.754	1.691–1.816
Binge	179	27.88	4.564	4.348–4.789
Male	374	100		
Abstainers	47	12.57	–	–
Moderate-drinkers	240	64.17	2.0	1.910–2.085
Binge-drinkers	87	23.26	5.138	4.847–5.429
**% drinking pattern**				
Drinkers	327	87.43	2.795	2.620–2.971
Moderate	240	73.39	2.0	1.910–2.085
Binge	87	26.61	5.138	4.847–5.429
Female	385	100		
Abstainers	70	18.18	–	–
Moderate-drinkers	223	57.92	1.457	1.385–1.529
Binge-drinkers	92	23.90	4.303	3.740–4.303
**% drinking pattern**				
Drinkers	315	81.82	2.243	2.076–2.410
Moderate	223	70.79	1.457	1.385–1.529
Binge	92	29.21	4.303	3.740–4.303

AUDIT-C, Alcohol Use Disorder Identification Test-C; n, number; CI, confidence interval.

**Figure 1:**
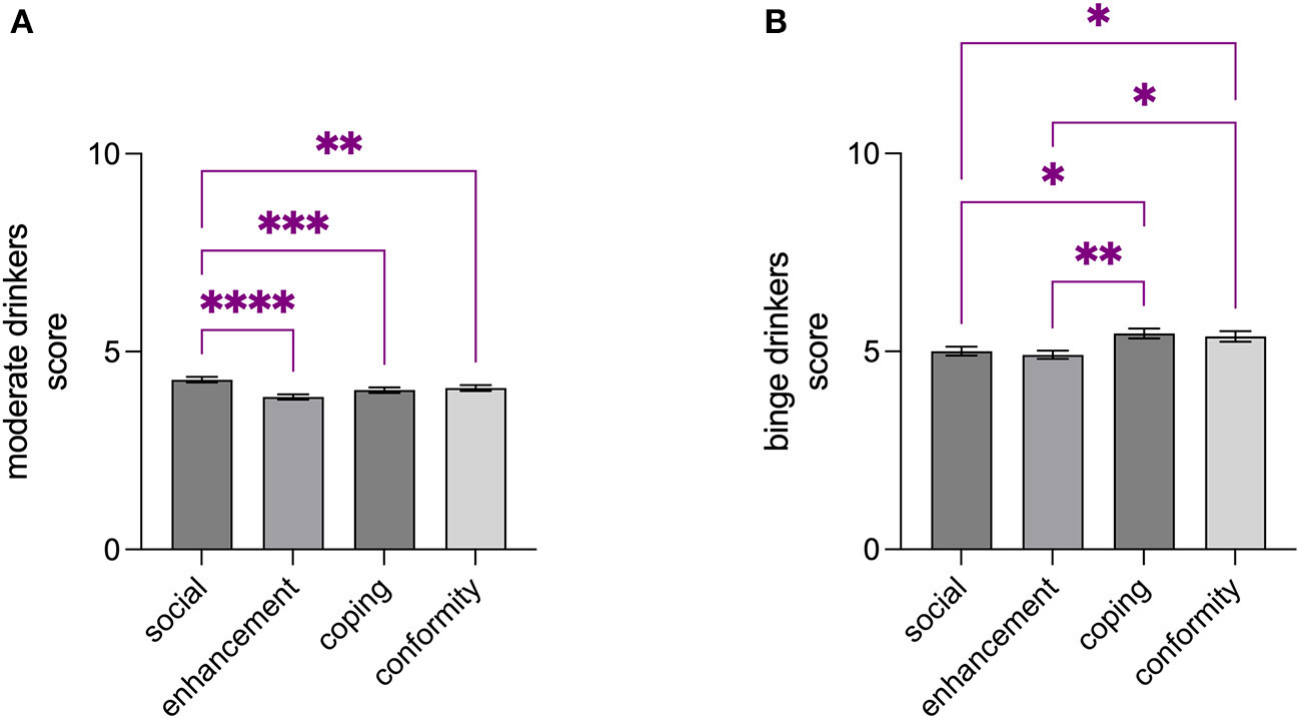
Drinking motives differently characterized moderate- and binge drinkers. Social motive score was found to be higher than enhancement-, coping-, and conformity scores among moderate drinkers **(A)**, while coping and conformity motive scores were found to be higher than social- and enhancement scores **(B)**. Data are shown as the mean ± SEM. *p < 0.05; **p < 0.01; ***p < 0.001; ****p < 0.0001.

Significant differences were detected among the drinking motives studied within binge- and moderate drinkers groups: the Kruskal-Wallis analysis showed a significant *P*-value for moderate drinkers (KW = 32.70; *p* < 0.0001), with social motive score being higher than enhancement (*Z* = 5.527, *p* < 0.0001), coping (*Z* = 3.936, *p* = 0.0005), and conformity (*Z* = 3.620, *p* = 0.0018) (Figure 1A); on the other hand, the analysis indicated a significant *P*-value for binge drinkers (KW = 18.36, *p* = 0.0004), with coping motive score being higher than social (*Z* = 3.001, *p* = 0.0161) and enhancement (*Z* = 3.376, *p* = 0.0044) and conformity motive score being higher than social (*Z* = 2.639, *p* = 0.0498) and enhancement (*Z* = 3.014, *p* = 0.0155) (Figure 1B). No significant differences were found when comparing the drinking motives of male and female binge-drinkers (coping: *U* = 3,824, *p* = 0.6016; conformity: *U* = 3,833, *p* = 6,207; enhancement: *U* = 3,864, p = 0.6823; social: *U* = 3,926, p = 0.8204; Table 2).

**Table 2:** Differences between male and female binge drinkers on drinking motives.

**Factor**	**Males**	**Females**	**Test**
	**(*n* = 87)**	**(*n* = 92)**	**Mann-Whitney** **test**
	**M (SD)**	**M (SD)**	
**Drinking motives questionnaire**			
Coping	5.45 (1.67)	5.48 (1.67)	*U* = 3,824 n.s.
Conformity	5.48 (1.73)	5.26 (1.77)	*U* = 3,833 n.s.
Enhancement	4.99 (1.34)	4.83 (1.44)	*U* = 3,864 n.s.
Social	5.06 (1.52)	4.96 (1.51)	*U* = 3,926 n.s.

n.s., not significant.

When students were stratified by alcohol drinking pattern, the occurrence of clinical syndromes and personality disorders—according to MCMI-III-, STAI-S/T-, CD-RISC-25- and BASIC-SE scores—was assessed. Significant differences among the three groups were shown. In particular, the Chi-square test revealed that the relationships between ABD and clinical syndromes, namely Anxiety (χ^2^ = 5.953, df = 1, *p* = 0.0147), Thought Disorder (χ^2^ = 4.909, df = 1, *p* = 0.0267), and Major Depression (χ^2^ = 4.372, df = 1, *p* = 0.0365), were significant. Moreover, the association between ABD and Depressive- (χ^2^ = 6.516, df = 1, *p* = 0.0107), Compulsive- (χ^2^ = 4.669, df = 1, *p* = 0.0307), Passive-Aggressive (Negativistic)- (χ^2^ = 8.293, df = 1, *p* = 0.0040), Schizotypal- (χ^2^ = 4.715, df = 1, *p* = 0.0299), Borderline- (χ^2^ = 10.76, df = 1, *p* = 0.0010), and Paranoid- (χ^2^ = 3.596, df = 1, *p* = 0.0579) personality disorders was found to be significant (Table 3). In addition, state- and trait anxiety, low resilience and self-esteem were significantly associated with AUDIT-C (STAI-S: χ^2^ = 14.49, df = 1, *p* = 0.0001; STAI-T: χ^2^ = 9.471, df = 1, *p* = 0.0021; CD-RISC-25: χ^2^ = 14.49, df = 1, *p* = 0.0001; BASIC-SE: χ^2^ = 9.471, df = 1, *p* = 0.0021; Table 3). Interestingly, the logistic regression analysis revealed that the AUDIT-C score was a relevant predictor of psychopathological traits in adolescents, as shown in Table 3.

**Table 3:** Psychopathological traits and alcohol drinking pattern, and predictive relation of AUDIT-C on MCMI-III, STAI-S/T, CD-RISC-25, and BASIC-SE scores.

**Psychopathological profile**
**Factor**	**Abstainers (*n* = 117)** **Frequency (%)**	**Moderate drinkers (*n* = 437)** **Frequency (%)**	**Binge drinkers (*n* = 171)** **Frequency (%)**	**TEST** **Prediction by AUDIT-C** **Simple Logistic Regression**
	**Chi Square of Pearson vs. ABSTAINERS**			
**MCMI-III**				
**Clinical syndromes scales**				
Anxiety	14 (11.97)	66 (15.10)	40 (23.39)	β1 = 1.164^**^
			χ^2^ = 5.953^*^	
Somatoform	–	–	2 (1.17)	β1 = 1.458 n.s.
			χ^2^ = 1.378 n.s.	
Bipolar: Maniac	3 (2.56)	12 (2.75)	10 (5.85)	β1 = 1.120 n.s.
			χ^2^ = 1.738 n.s.	
Dysthymia	2 (1.71)	10 (2.29)	9 (5.26)	β1 = 1.172 n.s.
			χ^2^ = 2.388 n.s.	
Alcohol dependence	0 (0)	0 (0)	0 (0)	
			–	
Drug dependence	–	1 (0.23)	4 (2.34)	β1 = 1.768^**^
			χ^2^ = 2.775	
Post-traumatic stress disorder	–	2 (0.46)	5 (2.92)	β1 = 1.538^**^
			χ^2^ = 3.481	
Thought disorder	–	5 (1.14)	7 (4.09)	β1 = 1.304^*^
			χ^2^ = 4.909^*^	
Major depression	4 (3.42)	9 (2.06)	17 (9.94)	β1 = 1.203^*^
			χ^2^ = 4.372^*^	
Delusional disorder	4 (3.42)	21 (4.81)	12 (7.02)	β1 = 1.045 n.s.
			χ^2^ = 1.715 n.s.	
**Personality disorders scales**				
Schizoid	6 (5.13)	2,910 (2.5)	7 (4.09)	β1 = 1.024 n.s.
			χ^2^ = 0.1725 n.s.	
Avoidant	9 (7.69)	36 (8.24)	13 (7.60)	β1 = 0.9392 n.s.
			χ^2^ = 0.0008 n.s.	
Depressive	6 (5.13)	38 (8.70)	25 (14.62)	β1 = 1.156^*^
			χ^2^ = 6.516^*^	
Dependent	9 (7.69)	33 (7.55)	17 (9.94)	β1 = 1.046 n.s.
			χ^2^ = 0.4279 n.s.	
Histrionic	7 (5.98)	20 (4.58)	6 (3.51)	β1 = 0.9808 n.s.
			χ^2^ = 0.9866 n.s.	
Narcisistic	28 (23.93)	79 (18.08)	38 (22.22)	β1 = 1.034 n.s.
			χ^2^ = 0.1149 n.s.	
Antisocial	–	1 (0.23)	4 (2.34)	β1 = 1.693^**^
			χ^2^ = 2.775	
Aggressive (Sadistic)	1(0.85)	9 (2.06)	4 (2.34)	β1 = 1.088 n.s.
			χ^2^ = 0.8974 n.s.	
Compulsive	5 (4.27)	7 (1.60)	1 (0.58)	β1 = 0.6188 n.s.
			χ^2^ = 4.669^*^	
Passive-Aggressive (Negativistic)	7 (5.98)	49 (11.21)	30 (17.54)	β1 = 1.219^**^
			χ^2^ = 8.293^**^	
Self-defeating	6 (5.13)	27 (6.18)	13 (7.60)	β1 = 0.9751 n.s.
			χ^2^ = 0.6901 n.s.	
Schizotypal	1 (0.85)	9 (2.06)	10 (5.85)	β1 = 1.254^*^
			χ^2^ = 4.715^*^	
Borderline	3 (2.56)	25 (5.72)	24 (14.04)	β1 = 1.322^****^
			χ^2^ = 10.76^**^	
Paranoid	2 (1.71)	17 (3.89)	11 (6.43)	β1 = 1.084 n.s.
			χ^2^ = 3.596	
**STAI-S/T**				
State anxiety	26 (22.22)	219 (50.00)	150 (87.50)	β1 = 2.349^**^
			χ^2^ = 14.49^***^	
Trait anxiety	26 (22.22)	291 (66.67)	128 (75.00)	β1 = 1.424 n.s.
			χ^2^ = 9.471^**^	
**CD-RISC-25**				
Low resilience	26 (22.22)	146 (33.33)	150 (87.50)	β1 = 0.2021^***^
			χ^2^ = 14.49^***^	
**BASIC-SE**				
Low self-esteem	26 (22.22)	219 (50.00)	128 (75.00)	β1 = 0.6450^*^
			χ^2^ = 9.471^**^	

* *p* < 0.05;

** *p* < 0.01;

*** *p* < 0.001;

**** *p* < 0.0001;

n.s., not significant; MCMI-III, Millon Clinical Multiaxial Inventory-Third Ed; STAI-S/T, State-Trait Anxiety Inventory-State; CDR-25, Connor-Davidson Resilience Scale; BASIC-SE, Basic Self-Esteem Scale.

**Figure 2:**
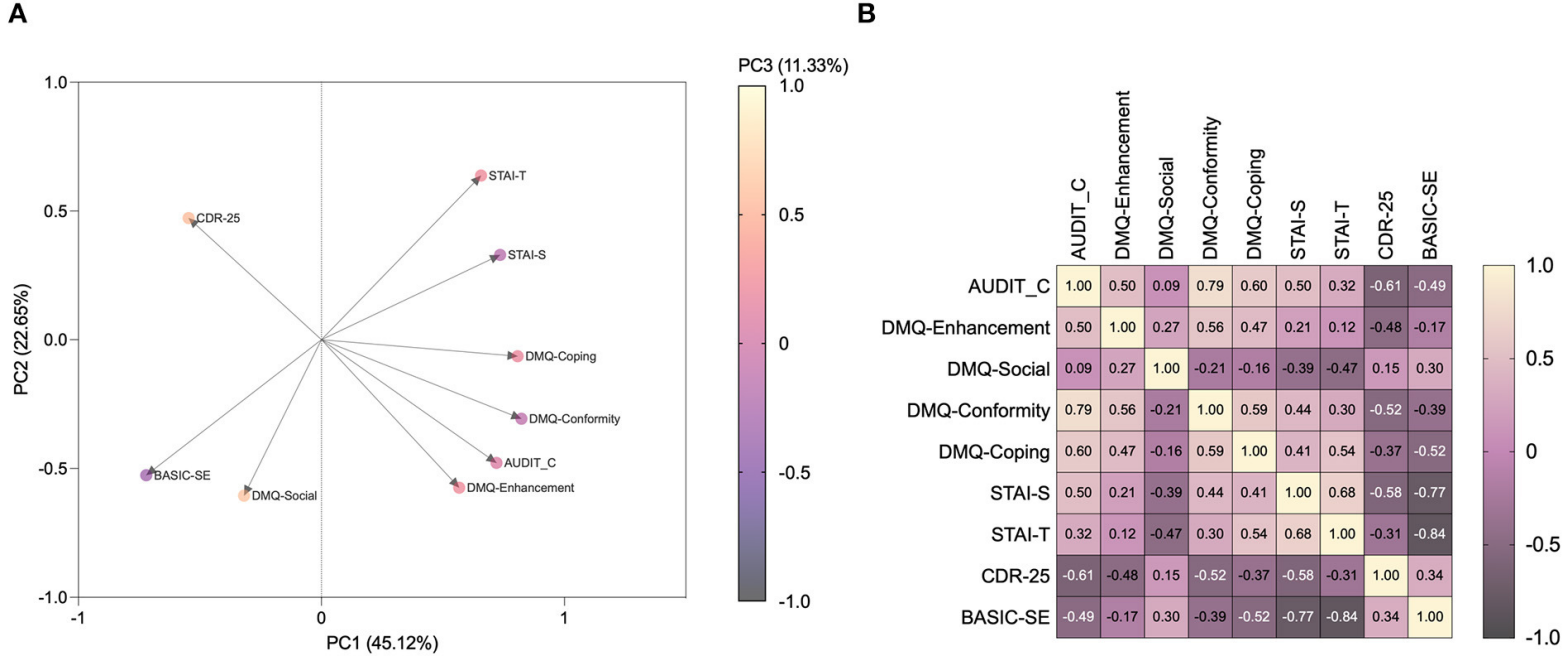
Anxiety, resilience, self-esteem, and drinking motives correlate with alcohol drinking patterns, neurovegetative and endocrine response. Loading-factor graph of principal component analysis **(A)** and the correlation matrix **(B)** show how alcohol drinking pattern and motives co-variate and correlate with anxiety-, resilience- and self-esteem levels observed in high school students. AUDIT-C, Alcohol Use Disorder Identification Test-C; DMQ, Drinking Motives Questionnaire-Revised Short Form; STAI-S/T, State-Trait Anxiety Inventory-State; CDR-25, Connor-Davidson Resilience Scale; BASIC-SE, Basic Self-Esteem Scale.

When all data were pooled in PCA, 79.10% of the overall variance was explained by the first three components. The main component, corresponding to 45.12% of the variance, was characterized by six of nine variables on the positive side, with the exception of CD-RISC-25-, BASIC-SE-, and DMQ-R-SF-Social scores (Figure 2A). The correlation matrix showed that AUDIT-C is positively associated with DMQ-R-SF-Enhancement (*r* = 0.499, *p* = 0.0019), DMQ-R-SF-Conformity (*r* = 0.792, *p* < 0.0001), and DMQ-R-SF-Coping (*r* = 0.603, *p* = 0.0001), STAI-S (*r* = 0.499, *p* = 0.0004), STAI-T (*r* = 0.321, *p* = 0.0291), negatively associated with CD-RISC-25 (*r* = −0.615, *p* < 0.0001), and BASIC-SE (*r* = −0.495, *p* = 0.0005), while non significantly associated with DMQ-R-SF-Social (*r* = 0.086, *p* = 0.6193). When anxiety, resilience, and self-esteem were assessed for the correlation to drinking motives, STAI-S was shown to be positively associated with DMQ-R-SF-Conformity (*r* = 0.441, *p* = 0.007), and DMQ-R-SF-Coping (*r* = 0.408, *p* = 0.0135) and negatively with DMQ-R-SF-Social (*r* = −0.387, *p* = 0.0197), Besides, STAI-T was found to be positively associated with DMQ-R-SF-Coping (*r* = 0.545, *p* = 0.0006), while negatively with DMQ-R-SF-Social (*r* = −0.466, *p* = 0.0042). On the other hand, CD-RISC-25 was negatively correlated to DMQ-R-SF-Enhancement (*r* = −0.478, *p* = 0.0032), DMQ-R-SF-Conformity (*r* = −0.523, *p* = 0.0011), and DMQ-R-SF-Coping (*r* = −0.374, *p* = 0.0247). Notably, BASIC-SE was negatively associated with DMQ-R-SF-Conformity (*r* = −0.387, *p* = 0.0197), and DMQ-R-SF-Coping (*r* = −0.518, *p* = 0.0012). Furthermore, state anxiety was negatively correlated to mental wellbeing protective factors, namely resilience (CD-RISC-25: *r* = −0.579, *p* < 0.0001) and self-esteem (BASIC-SE: *r* = −0.771, *p* < 0.0001). As so, STAI-T was negatively correlated to CD-RISC-25 (*r* = −0.310, *p* = 0.0362), and BASIC-SE (*r* = −0.844, *p* < 0.0001). On the other hand, resilience and self-esteem were found to be positively correlated one to the other (CD-RISC-25 to BASIC-SE: *r* = 0.338, *p* = 0.0214) (Figure 2B).

### Anxiety-like phenotype and stress reactivity in the rat model of binge-like alcohol exposure

**Figure 3:**
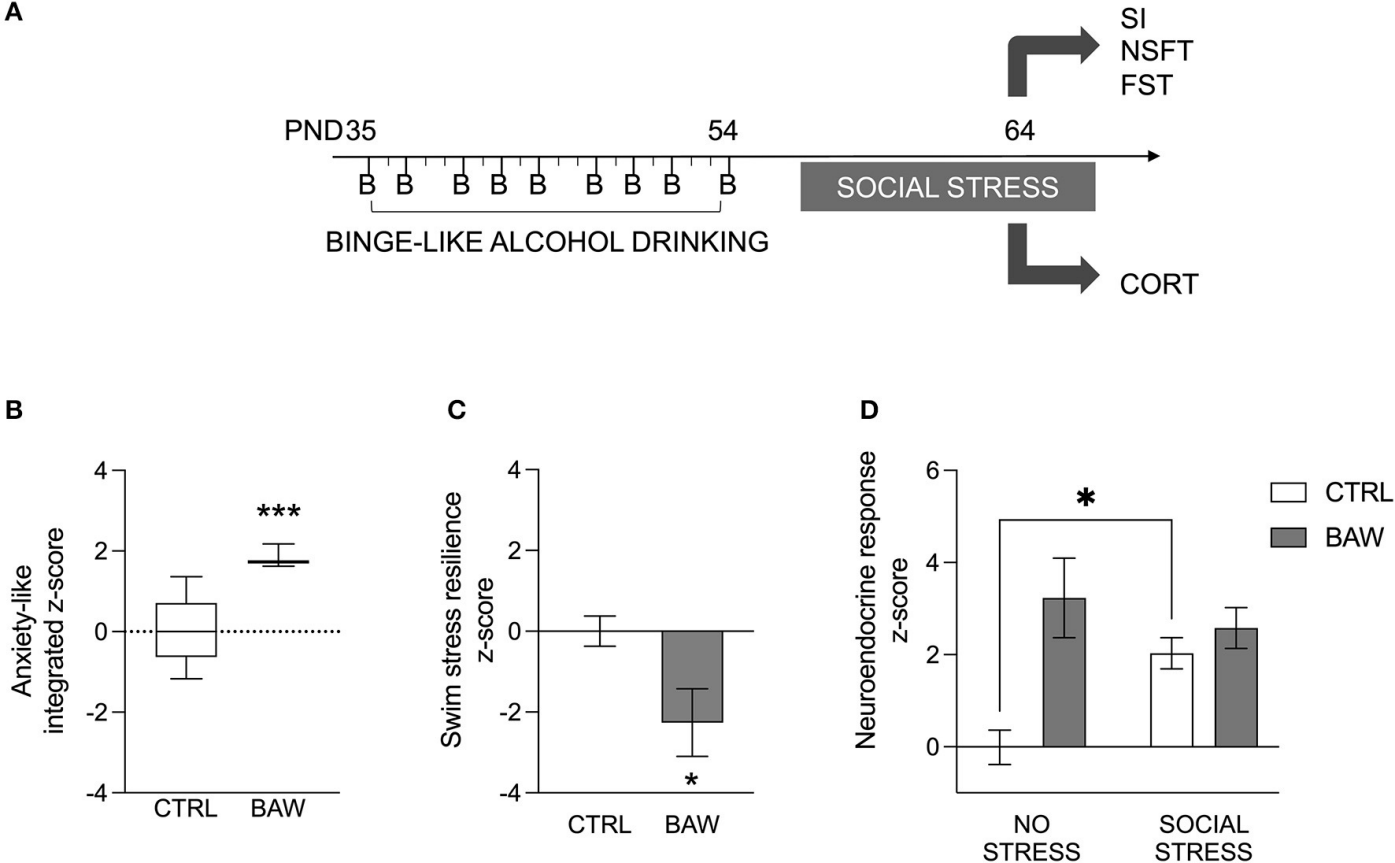
Anxiety-like phenotype and stress reactivity in the rat model of binge-like alcohol exposure. Alcohol binge drinking during adolescence was modeled in rats in order to assess anxiety-like phenotype and stress reactivity **(A)**. Altered anxiety-like phenotype and stress reactivity in the rat model of binge-like alcohol exposure. Rats exposed to binge-like alcohol during adolescence displayed a higher integrated *z*-score referring both to anxiety-like behavior under social or novel circumstances than CTRL rats **(B)**. When the active coping to the swim stress was evaluated as a measure of resilience, BAW rats displayed a lower score than the CTRL group **(C)**. Moreover, binge-like alcohol exposure altered the neuroendocrine stress response in adolescent rats. In detail, when CTRL rats were exposed to social stress, they displayed increased CORT serum levels, with respect to basal levels; on the other hand, no significant increase was observed in BAW rats **(D)**. Data are shown as the mean ± SEM. *p < 0.05; ***p < 0.001. CTRL, control group; BAW, binge-like alcohol-exposed rats; SI, social interaction test; NSFT, novelty suppressed feeding test; FST, forced swimming test; CORT, corticosterone.

Alcohol binge drinking during adolescence was modeled in rats in order to assess anxiety-like phenotype and stress reactivity (Figure 3A). The analysis of the anxiety-like phenotype in the rat model indicated that rats exposed to binge-like alcohol during adolescence displayed a higher integrated *z*-score referring both to anxiety under social circumstances and novelty-induced anxiety-like behavior than CTRL rats (*p* = 0.0006) (Figure 3B). In addition, when the active coping with the swim stress was evaluated as a measure of resilience, BAW rats showed a significant decrease in the resilience score compared with the CTRL group (*p* = 0.0262) (Figure 3C). Moreover, binge-like adolescent alcohol exposure altered the neuroendocrine stress response in rats [BAW: F_(1, 24)_ =11.97, *p* = 0.0020; stress: F_(1, 24)_ = 1.616, *p* = 0.2159; interaction: F_(1, 24)_ = 6.066, *p* = 0.0213]. In detail, when CTRL rats were exposed to the social stress, they displayed increased relative CORT serum levels with respect to the no-stress exposed CTRLs (*t* = 2.640, df = 24.00, *p* = 0.0287); on the other hand, no significant increase was observed in BAW rats (*t* = 0.8428, df = 24.00, *p* = 0.8153) (Figure 3D).

## Discussion

The current research was undertaken to explore the alcohol-drinking habits, the main motives to drink, and the psychological profiles of high school students in Southern Italy. Then, we investigated, in the variable-protected environment of the lab setting, whether young rats with a history of binge-like alcohol exposure during adolescence developed a vulnerable endophenotype for emotional dysregulation and negative affective state.

Our data shows that of the 759 students surveyed, 84.59% consumed alcohol and, among them, 27.88% did it in a binge-like manner. Accordingly, recent findings on alcohol drinking in Mediterranean areas report a high prevalence of the binge pattern in a 15–24-year old population in Cyprus and Malta (26%), as well as Portugal (28%), Spain and Greece (34%) (44), which align with the Nordic rate (45–47). This is in agreement with an Italian study showing that 90% of the questioned 18–24-year-old students reported drinking alcohol and 30% were binge drinkers; however, the percentage was different in the two sexes with male and female binge drinkers accounting for, respectively, 41.5 and 31.4% (48). In contrast, our data highlights a similar ABD prevalence in both sexes, suggesting that the male-female gradient is gradually decreasing if not reverting in Italy (49). Yet, given the unique gender gap in alcohol metabolism and, thus, vulnerability to neurotoxicity, gender-tailored intervention and prevention should be planned (50).

Previous research indicates that different patterns of consumption are linked to different alcohol drinking motives (8, 51). When the motives underlying drinking behavior were explored in our sample, moderate drinkers indicated sociality as the main motive, in agreement with previous studies investigating why and how much young people drink (4, 52). Indeed, during adolescence, social gathering becomes increasingly important and alcohol is perceived as a help to gain popularity if consumed in a moderate pattern (8). On the other hand, coping with stress and conformity significantly characterized the young binge drinkers, more than social and enhancement motives, independently from their sex. Coping motives have been previously associated to alcohol abuse and alcohol-related problems (53); rather, conformity implies the alignment to the most popular drinking trend in social context, thus contributing to the spreading of the “Nordic” binge drinking pattern among adolescents in the Mediterranean areas (54). Interestingly, when we related AUDIT-C score, which provides a measure of the risky drinking pattern, with motives a strong correlation was found for conformity and coping. Indeed, adolescence is characterized by high psychological and social vulnerability; thus, similar to the tension reduction alcohol use hypothesis (55), the emotion regulation theory of binging disorders posits that binging offers an escape from aversive feelings related to stressing environmental situations (56). However, AUDIT-C score also correlated with enhancement, indicating that to be “on-a-high” drives to the binge drinking pattern.

Previous research assessed the prevalence of certain psychopathological traits in adult alcohol abusers, highlighting a “dual pathology”, i.e., the development of a mental disorder and a pathological dependence that affect each other, in a not established causal relationship (57). Indeed, high rates of comorbidity have been observed between alcohol misuse and personality disorders of the impulse dyscontrol spectrum, such as antisocial and borderline personality disorder (58–62); alcoholic patients displayed higher score than control subjects on measures of the negativistic trait with depressive and anxiety symptomatology (63, 64); moreover, evidence from young population showed how high levels of alcohol drinking are related to low levels of self-esteem (65, 66). The analysis of the MCMI-III showed that adolescent binge-drinking students display a higher percentage of clinical syndromes such as anxiety, thoughts disorder, and major depression, and of personality disorders including depressive, compulsive- and passive-aggressive-, schizotypal- and borderline trait, than abstainers. Notably, the STAI test confirms the presence of a higher percentage of state and trait anxiety in bingers than abstainers. Accordingly, students with a marked ABD pattern were found to score almost twice as high as the general adult population on the stress perception scale, indicating a high level of emotionality (67).

Growing evidence suggests that ABD in adolescence correlates with higher tonic levels of heart rate and cortisol, which indicate a hypersensitivity of the brain stress system (68). Interestingly, in these subjects HPA axis activity assumes a predictive value for problematic alcohol drinking behavior (69), as shown by higher odds of ABD on particularly stressing days (70), suggesting a higher vulnerability, and a lower resilience to coping with stress.

While the occurrence of the psychopathological identikit is considered a vulnerability factor able to contribute to the maintenance of problematic alcohol drinking (71), resilience and self-esteem refer to protective factors for physical and mental health, meaning a positive adaptation and development in the context of significant threats (72). Indeed, bingers were characterized by higher rates of low resilience and self-esteem. Epidemiologic studies indicate a clear association between low resilience and increased addictive behaviors, as well as high levels of alcohol intake and low self-esteem in young people (65, 73, 74). Notably, AUDIT-C emerged as a relevant predictor of the occurrence of discrete clinical syndromes and personality disorders, as well as of low resilience and self-esteem: the increase in AUDIT-C score enhances the likelihood of psychopathological traits. Likewise, we observed that resilience and self-esteem displayed a negative correlation not only with the alcohol drinking pattern but also with anxiety state/trait score. Therefore, our data suggest that low resilience is able to trigger the flywheel relationship between stress, anxiety, and alcohol intake, increasing the risk of developing problematic alcohol use (75, 76). On the other hand, alcohol “peaks-and-drops” typical of the cycle of intoxication and abstinence of ABD can impact the functioning of brain regions involved in behavioral adaptation, stress coping, emotional control, promoting the occurrence of a vulnerable psychopathological phenotype for negative affect (77).

Whether the characteristic psychopathological profile results from, or triggers ABD is hard to say.

Interestingly, early reports from this group show an opposite co-variance between stress-triggered anxiety-like behavior in alcohol binge-like exposed adolescent rats and ethologically relevant correlates of resilience, such as positive social outcomes (30). Indeed, animal models enable appropriate isolation of individual variables and help identify the relationship between environmental factors and modifications in physiological responses (78–81). An abnormal reactivity to novel environmental challenges and impairment in reward/aversion processing characterized late adolescent rats with a history of alcohol binge-like exposure. As social avoidance reflects anxiety-like behavior under social circumstances (40), hyponeophagia is generally considered an anxiety-like feature observed following repeated alcohol consumption in rats (82). At the same time, reduced motivational salience associated with natural rewarding stimuli, i.e., food and socializing in our experimental conditions, is specifically reported both in social-anxiety disorders and alcohol abuse (83). This data suggests that altered motivation/aversion processing and abnormal behavioral reactivity reflect the occurrence of a negative affective state in alcohol binge-like exposed rats. Moreover, these rats displayed increased immobility in the forced swim stress test. A reduction in the attempts to escape from water can be interpreted as behavioral despair, which is suggestive of reduced resilience to the aversive environment as a result of alcohol binge-like exposure in adolescence. These results were associated with allostatic adaptations in stress regulatory pathways, as shown by higher basal CORT levels, and a blunted response to the psychosocial challenge. Previous evidence from this group demonstrates that alcohol binge-like exposure during pubertal development is associated with increased circulating plasma CORT levels and CRH expression, in both hypothalamic and extra-hypothalamic circuits, suggesting that the disruption of glucocorticoid feedback plays a role in the development of negative emotional states (30). Notably, although this preclinical study did not include female rats, due to the unreliability of male aggressors toward young females, and the need for irreversible hypothalamic lesions in female aggressors (84), our current research is ongoing to evaluate the relationship between binge-like alcohol in adolescence and response to social stress in refined sex-specific paradigms.

Indeed, more effort is needed to crack the chicken-or-egg question on binge alcohol drinking in adolescence and the occurrence of psychopathological traits and clinical syndromes. However, although reciprocal influences and shared risk factors have been proposed, recent longitudinal studies and meta-analyses show that the presence of anxiety and depression in adolescence does not consistently predict later pattern and binge drinking (85–87). On the other hand, our evidence suggests that binge-like alcohol exposure in adolescence induces the occurrence of negative affect, emotional dysregulation, and aberrant stress response, in those that do not present this phenotype ab initio.

## Conclusions

The prevalence of ABD among high school students from Southern Italy, sustained by coping, enhancement, and conformity motives, is aligned with the Nordic rate. Thus, drinking “too-much-too-fast” is erasing the differences in drinking habits among EU regions. We also highlight how ABD is related to a greater likelihood of presenting a psychopathological profile characterized by emotional dysregulation and negative affect, together with increased vulnerability to stressful events. Overall, the preclinical behavioral and endocrine data indicate a close relationship between alcohol binge-like drinking and the occurrence of anxiety-like- and maladaptive response to stress that may have a translational relevance. Indeed, although it does not fully answer the “chicken-or-egg” question, our data emphasize the importance of contrasting ABD as a primary prevention goal in the youth population.

## Data availability statement

The raw data supporting the conclusions of this article will be made available by the authors, without undue reservation.

## Ethics statement

The studies involving human participants were reviewed and approved by Ethics Committee Palermo 1. The patients/participants provided their written informed consent to participate in this study. The animal study was reviewed and approved by Organismo preposto al benessere degli animali (OPBA).

## Author contributions

VC and FP: methodology, formal analysis, investigation, data curation, and writing—original draft preparation. GM: methodology, formal analysis, investigation, and data curation. GL: investigation. GP: methodology. AB: formal analysis, investigation, and writing—reviewing and editing. CC: conceptualization, methodology, writing—reviewing and editing, supervision, project administration, and funding acquisition. All authors contributed to the article and approved the submitted version.

## Funding

This work was supported by the European Foundation for Alcohol Research-ERAB (grant number: EA 16 42, to CC); and Fondazione Zardi Gori (post-doctoral fellowship to AB).

## Conflict of interest

The authors declare that the research was conducted in the absence of any commercial or financial relationships that could be construed as a potential conflict of interest.

## Publisher's note

All claims expressed in this article are solely those of the authors and do not necessarily represent those of their affiliated organizations, or those of the publisher, the editors and the reviewers. Any product that may be evaluated in this article, or claim that may be made by its manufacturer, is not guaranteed or endorsed by the publisher.
